# Precipitation at Room Temperature as a Fast and Versatile Method for Calcium Phosphate/TiO_2_ Nanocomposites Synthesis

**DOI:** 10.3390/nano11061523

**Published:** 2021-06-09

**Authors:** Ina Erceg, Atiđa Selmani, Andreja Gajović, Borna Radatović, Suzana Šegota, Marija Ćurlin, Vida Strasser, Jasminka Kontrec, Damir Kralj, Nadica Maltar-Strmečki, Rinea Barbir, Barbara Pem, Ivana Vinković Vrček, Maja Dutour Sikirić

**Affiliations:** 1 Laboratory for Biocolloids and Surface Chemistry, Ruđer Bošković Institute, Bijenička Cesta 54, 10000 Zagreb, Croatia; ierceg@irb.hr (I.E.); aselmani@irb.hr (A.S.); ssegota@irb.hr (S.Š.); Vida.Strasser@irb.hr (V.S.); 2 Laboratory for Energy Conversion Materials and Sensors, Ruđer Bošković Institute, Bijenička Cesta 54, 10000 Zagreb, Croatia; gajovic@irb.hr; 3 Institute of Physics, Bijenička Cesta 46, 10000 Zagreb, Croatia; bradatovic@ifs.hr; 4 Department of Histology and Embryology, School of Medicine, University of Zagreb, Šalata 3, 10000 Zagreb, Croatia; marija.curlin@mef.hr; 5 Laboratory for Precipitation Processes, Ruđer Bošković Institute, Bijenička Cesta 54, 10000 Zagreb, Croatia; lugaric@irb.hr (J.K.); kralj@irb.hr (D.K.); 6 Laboratory for Magnetic Resonances, Ruđer Bošković Institute, Bijenička Cesta 54, 10000 Zagreb, Croatia; nstrm@irb.hr; 7 Institute for Medical Research and Occupational Health, Ksaverska Cesta 2, 10000 Zagreb, Croatia; rbarbir@imi.hr (R.B.); bpem@imi.hr (B.P.); ivinkovic@imi.hr (I.V.V.)

**Keywords:** calcium phosphates, TiO_2_ nanoplates, TiO_2_ nanowires, precipitation, simulated body fluid, reactive oxygen species, hemocompatibility

## Abstract

The constantly growing need for advanced bone regeneration materials has motivated the development of calcium phosphates (CaPs) composites with a different metal or metal-oxide nanomaterials and their economical and environmentally friendly production. Here, two procedures for the synthesis of CaPs composites with TiO_2_ nanoplates (TiNPl) and nanowires (TiNWs) were tested, with the immersion of TiO_2_ nanomaterials (TiNMs) in corrected simulated body fluid (c-SBF) and precipitation of CaP in the presence of TiNMs. The materials obtained were analyzed by powder X-ray diffraction, spectroscopic and microscopic techniques, Brunauer–Emmett–Teller surface area analysis, thermogravimetric analysis, dynamic and electrophoretic light scattering, and their hemocompatibility and ability to induce reactive oxygen species were evaluated. After 28 days of immersion in c-SBF, no significant CaP coating was formed on TiNMs. However, the composites with calcium-deficient apatite (CaDHA) were obtained after one hour in the spontaneous precipitation system. In the absence of TiNMs, CaDHA was also formed, indicating that control of the CaP phase formed can be accomplished by fine-tuning conditions in the precipitation system. Although the morphology and size of crystalline domains of CaDHA obtained on the different nanomaterials differed, no significant difference was detected in their local structure. Composites showed low reactive oxygen species (ROS) production and did not induce hemolysis. The results obtained indicate that precipitation is a suitable and fast method for the preparation of CaPs/TiNMs nanocomposites which shows great potential for biomedical applications.

## 1. Introduction

The modern way of life and population aging results in an increased frequency of chronic diseases, among which the most significant are hard tissue (bone and teeth) chronic diseases. These diseases occupy a special role due to their presence in all age groups, significantly reduced patients’ quality of life, and influence upon society in general [1,2,3]. In many cases, the only treatment for bone diseases or injuries is implantation which itself is connected with the risks of failure. These problems encourage the development of new, innovative implant materials. Such materials should be produced in environmentally friendly ways and be of low cost, available to as many patients as possible and, therefore, produce additional values for established healthcare systems. Among different classes of material of interest, calcium phosphates (CaPs)/TiO_2_ composites are of special interest because of the complementary advantages that these two materials possess [4,5,6,7,8,9,10].

Calcium phosphates are the first choice for bone implant materials since the main inorganic component of the bones is biological apatite, i.e., non-stoichiometric poorly crystalline, calcium-deficient, sodium, magnesium- and carbonate hydroxyapatite [11]. Today, 13 different CaPs phases are known, out of which hydroxyapatite [HAP, Ca_10_(PO_4_)_6_(OH)_2_], calcium deficient apatite [CaDHA, Ca_10−*x*_(HPO_4_)*_x_*(PO_4_)_6−*x*_(OH)_2−*x*_, 0 < *x* < 1], octacalcium phosphate [OCP, Ca_8_(HPO_4_)_2_(PO_4_)_4_·5H_2_O], α and β tricalcium phosphates [α-, β-TCP, Ca_3_(PO_4_)_2_] and calcium hydrogenphosphate dihydrate [DCPD, CaHPO_4_·2H_2_O], are used in bone tissue engineering [11]. CaPs possess excellent biocompatibility and osteoconductivity. However, their major disadvantage is poor mechanical properties, which limit their application to non-load-bearing applications [12]. A likely solution to this problem is the development of CaPs’ composites with titanium, increasingly focusing on composites with different TiO_2_ nanomaterials (TiNMs) [7,8,13,14,15]. In addition to excellent mechanical properties and biocompatibility [16], TiO_2_ can improve composites’ anticorrosive properties [6,17], osteoblast adhesion [18], and potentially also antibacterial activity [19]. Besides biomedical applications, such composites are also of interest in photocatalysis [20] and environmental protection [21].

Different strategies have been employed for preparing the CaP/TiO_2_ composites in the form of powder, such as hydrothermal synthesis combined with a high gravity [20], microwave hydrothermal technique [22], in situ precipitation at higher temperatures [17], sintering of powder mixtures [5,6], high-energy ball milling [23] and the sol-gel method [8,24,25]. However, these methods use high temperatures or need a long reaction time and are not environmentally friendly [8,17]. Salarian et al. succeeded in improving the sol-gel procedure by supercritical fluid processing with carbon dioxide obtaining HAP nanoplates grown from the surface of one-dimensional titania nanorods [8], while Enayatti-Jazi et al. succeeded in preparing HAP/TiO_2_ nanocomposites by in situ precipitation at 70 °C [17].

Wet chemical synthesis at temperatures below 100 °C attracts special attention for the synthesis of such materials. The reasons lie in improved control of formed calcium phosphates’ phase and the possibility of incorporating bioactive molecules. In addition, low-temperature wet techniques are performed using simple equipment and are of low cost [26]. If performed at experimental conditions close to physiological, they mimic the hard tissues’ formation in the living organisms which is considered to be an additional advantage [26]. Strictly speaking, the biomimetic preparation involves immersing the substrates into simulated body fluid (SBF) [27,28] at room or physiological temperature. However, this procedure can be lengthy, which can be surpassed by increasing the concentration of SBF [29], by pre-treating the surface of the substrate [15], or by using other metastable calcium and phosphate ion-containing solution [30,31].

Surprisingly, the investigations of biomimetic preparation of CaP/TiO_2_ composites are scarce and mostly focused on determining in vitro bioactivity of TiO_2_ nanomaterials. Ruso et al. [7] have shown that amorphous calcium phosphate can be formed after just one day of soaking in SBF on decahedral anatase nanoparticles with a high content of reactive {100} facets. Recently our group has shown that TiO_2_ nanoparticles (TiNPs) and titanate nanotubes (TiNTs) show low ability to induce the formation of CaPs in corrected simulated body fluid (c-SBF) which was explained by their small negative zeta-potential [13]. To surpass this obstacle, we have applied an alternative method using TiNMs as a suspended substrate during precipitation of CaP from a precipitation system with reactant concentrations somewhat higher than physiological at 25 °C. Already within 1 h, the uniform CaDHA layer was formed on the surface of both TiNMs [13]. To the best of our knowledge, no biomimetic preparation of composites with other types of TiNM has been performed to date.

In this paper, we extended our approach and tested the biomimetic mineralization of TiO_2_ nanoplates (TiNPls) and TiO_2_ nanowires (TiNWs) in SBF and during CaPs precipitation in order to verify the applicability of the proposed precipitation synthetic method on TiNMs of different composition, surface structure, and morphology. As well, the potential of the CaP/TiNM nanocomposites obtained for biomedical application was tested by determining the acellular reactive oxygen species (ROS) generation and hemocompatibility.

## 2. Materials and Methods

### 2.1. Materials

Analytical grade chemicals sodium hydrogenphosphate (Na_2_HPO_4_), di-potassium hydrogen phosphate trihydrate (K_2_HPO_4_·3H_2_O), calcium chloride (CaCl_2_), sodium chloride (NaCl), potassium chloride (KCl), magnesium chloride hexahydrate (MgCl_2_·6H_2_O), sodium hydrogencarbonate (NaHCO_3_), sodium sulfate (Na_2_SO_4_), Tris-hydroxymethyl aminomethane ((HOCH_2_)_3_CNH_2_, Tris), hydrochloric acid (HCl), sodium hydroxide (NaOH), TiO_2_ nanoparticles, TiO_2_ P25 Degussa (75% anatase, 25% rutile), titanium tetraisopropoxide (TIIP), hydrofluoric acid (HF), 2′,7′-dichlorodihydrofluoresin diacetate (DCFH_2_-DA), methanol, Triton X-100, Polyethylene glycol 600 and Hemoglobin (Hb) standard were obtained from Sigma Aldrich, Darmstadt, Germany. In addition, 3-morpholinosydnonimine hydrochloride (SIN-1 hydrochloride) was obtained from Abcam, Cambridge, UK, Gibco DPBS from Thermofisher Scientific, Waltham, MA, USA, and Drabkin’s reagent from Randox, Crumlin, County Antrim, UK. Ultrapure water (UPW, conductivity 0.5 μS cm^−1^, Hydrolab HLP 10 UV, Straszyn, Poland) was used throughout.

### 2.2. Synthesis of TiO_2_ Nanomaterials

TiNPls were prepared by acid hydrothermal synthesis similar to the procedure by Han et al. [32] In short, HF was added dropwise to TIIP in the Teflon dish. The Teflon dish was placed in an autoclave and heated for 24 h at 180 °C. The formed product was filtered and washed with UPW water. To remove F^−^ ions, TiNPls were incubated with s 0.1 mol L^−1^ NaOH for 24 h, washed with UPW till the conductivity of mother liquor was lower than 10 μS cm^−1^. Afterward, the fluoride residues were additionally removed by calcination at 500 °C for 2 h [33].

TiNWs and titanate nanotubes were synthesized by alkaline hydrothermal synthesis as described by Selmani et al. [34]. TiO_2_ P25 particles were suspended in 10 mol L^−1^ NaOH solution by magnetic stirring and sonication for 2 h. The suspension obtained was transferred into a Teflon-lined autoclave and heated for 24 h at 180 °C to prepare TiNWs and 48 h at 146 °C to obtain titanate nanotubes. The TiNWs and titanate nanotube precipitates obtained were washed with UPW. Excess Na^+^ ions were removed by magnetically stirring TiNWs and titanate nanotubes in 0.1 mol L^−1^ HCl for 3 h. Subsequently, TiNWs were washed with UPW until the conductivity of mother liquor was lower than 10 μS cm^−1^, filtered, and dried at 100 °C for 8 h.

### 2.3. Mineralization of TiO_2_ Nanopalates (TiNPls) and Nanowires (TiNWs) in Simulated Body Fluid (SBF)

For preparation of c-SBF, the procedure described by Kokubo and Takadama was used [28]. The c-SBF contained 142 mM Na^+^, 5 mM K^+^, 1.5 mM Mg^2+^, 2.5 mM Ca^2+^, 147.8 mM Cl^−^, 4.2 mM ${\mathrm{HCO}}_{3}^{-}$, 1.0 mM ${\mathrm{HPO}}_{4}^{2-}$ and 0.5 mM ${\mathrm{SO}}_{4}^{2-}$ [28].

We immersed 10 mg of TiNPls or TiNWs in 1 mL of c-SBF and kept these in a thermostated water bath for 28 days at 25 °C. SBF was changed daily. Samples for powder X-ray diffraction (PXRD) characterization were collected after 1, 3, 7, 14, 21, and 28 d through 0.45 μm Millipore filter paper. Precipitates were washed 3 times with UPW and ethanol, dried in the stream of nitrogen, and kept in a desiccator until analysis. The morphology of the precipitates obtained after 28 days of immersion was visualized by scanning electron microscopy.

### 2.4. Mineralization of TiNPls and TiNWs by Spontaneous Precipitation of Calcium Phosphates

Stock solutions of CaCl_2_ and Na_2_HPO_4_ were prepared by dissolving the required amount of analytical grade chemicals, which were dried overnight in a desiccator over silica gel, in UPW. HCl was used to adjust the pH of the Na_2_HPO_4_ stock solution to 7.4. TiNMs stock suspensions were prepared by suspending the needed amount of powder in UPW using ultrasound. Precipitation was initiated by the simultaneous mixing of equal volumes of equimolar CaCl_2_ and Na_2_HPO_4_ solutions prepared by diluting the respective stock solution. The initial reactant concentrations in precipitation system were *c*(CaCl_2_) = *c*(Na_2_HPO_4_) = 4 · 10^−3^ mol dm^−3^ at pH = 7.4. To determine the influence of TiNMs, precipitation systems without the TiNMs (control system, CS) and containing TiNMs were prepared. Systems containing TiNMs were prepared by adding the required amount of their stock suspension in phosphate solution before mixing reactant solutions and readjusting pH when necessary. The precipitation experiments were carried out in a double-wall vessel at 25 ± 0.1 °C without additional stirring. The advancement of the precipitation was followed by monitoring pH changes (Metrohm 701 pH/Ion meter). Induction times (*t*_i_), i.e., times needed for the commencement of amorphous to crystalline phase transformation were determined from the obtained pH vs. time curves. Based on the determined *t*_i_, samples were filtered after 10 and 60 min through a 0.45 μm Millipore filter. The precipitates obtained were washed three times with UPW and ethanol. Subsequently, they were dried in the stream of nitrogen and kept in a desiccator until further analysis.

### 2.5. Characterization Methods

#### 2.5.1. Powder X-ray Diffraction (PXRD)

PXRD patterns of the precipitates were recorded on Panalytical Aeris Research Edition (Malvern Pananalytical, Malvern, Worcestershire, UK) in Bragg–Brentano geometry using CuK_α_ radiation. Angular scan range from 5° to 70° 2*θ* with a step size of 0.02° 2*θ* and a scan rate of 1° min^−1^ was used.

#### 2.5.2. Raman Spectroscopy (RS)

The micro-Raman spectra were recorded by a Horiba Jobin-Yvon T64000 system (Kyoto, Japan) equipped with a solid-state laser operating at 532.5 nm for excitation. The nominal laser power of 20 mW was focused by a 50× magnification objective lens (Olympus, Tokyo, Japan) on the samples.

#### 2.5.3. Brunauer–Emmett–Teller Surface Area Analysis (BET)

The specific surface area was determined by the multiple Brunauer–Emmett–Teller (BET) method (Gemini 2380, Micromeritics Norcross, GA, USA). Nitrogen gas was used as the adsorbate.

#### 2.5.4. Dynamic Light Scattering (DLS) and Electrophoretic Light Scattering (ELS) Measurements

The TiNMs’ size distribution and zeta potential were determined in c-SBF and anionic reactant solution by the dynamic and electrophoretic light scattering using a photon correlation spectrophotometer equipped with a 532 nm “green” laser (Zetasizer Nano ZS, Malvern Instruments, Worcestershire, UK). For dynamic light scattering (DLS) measurements, the intensity of scattered light was detected at the angle of 173°. The hydrodynamic diameter (*d*_h_), value at the peak maximum, was obtained from the size volume distribution function to avoid overestimation due to the scattering of larger particles. The zeta potential (*ζ*) was obtained from the measured electrophoretic mobility using the Henry equation and the Smoluchowski approximation. For each sample, measurements were repeated five times, and representative data are shown. Data processing was carried out using Zetasizer Software 7.13 (Malvern Instrument, Worcestershire, UK). All measurements were p at 25.0 ± 0.1 °C.

#### 2.5.5. Thermogravimetric Analysis (TGA)

Thermogravimetric analysis (TGA) measurements were dynamic (non-isothermal) runs performed in Mettler TG 50 thermobalance with a TC 10 TA processor (Mettler Toledo, Columbus, OH, USA). The samples were heated over the temperature range of 25–800 °C at a rate of 10 °C/min and under a dynamic air atmosphere flowing at 20 mL min^−1^. The weight loss was measured as a function of temperature.

#### 2.5.6. Electron Paramagnetic Resonance Spectroscopy (EPR)

The radiation induced radicals were used for electron paramagnetic resonance spectroscopy (EPR) analysis. All samples were irradiated, in the air, using a ^60^Co gamma-ray source of the Ruđer Bošković Institute [35], to a cumulative dose of 25 kGy. The dose of 25 kGy was chosen following the International Atomic Energy Agency (IAEA) recommendation [36] of using 25 kGy as the standard dose for terminal sterilization of medical products, including bone allografts. EPR measurements were performed on a Varian E-109 X-band (~9.3 GHz) spectrometer (Palo Alto, Santa Clara, CA, USA) combined with a Bruker ER 041 XG microwave bridge. All EPR measurements were performed at 25.0 ± 0.1 °C using the Bruker ER4111VT (Billerica, MA, USA) variable temperature unit. To calibrate the spectrometer magnetic field, the Mn^2+^/MgO standard reference sample was used. All EPR spectra were normalized by sample mass. The custom-built program in MATLAB (The MathWorks Inc., Natick, MA, USA) using the EasySpin program package [37] was used for running the simulations of the experimentally obtained EPR spectra.

#### 2.5.7. Scanning Electron Microscopy (SEM)

The morphology of the investigated materials was determined by a field emission scanning electron microscope (FE-SEM; JEOL JSM-7000F microscope, Tokyo, Japan) and by a tungsten filament electron microscope (SEM; TESCAN VEGA 3 microscope, Fuveau, France). A required amount of powder was put on a sample holder covered with carbon glue and the excess powder was removed by gentle nitrogen gas flow.

#### 2.5.8. Transmission Electron Microscopy and Selected Area Electron Diffraction (TEM/SAED)

Transmission electron microscopy (TEM) images were recorded on LaB Philips CM200 TEM (Philips Electronics, Eindhoven, The Netherlands) and Jeol JEM1010 (Tokyo, Japan) microscopes operated at 80 kV. The drop of the suspension was put on the copper grid covered with the hollow Formvar membrane. The excess solution was removed by filter paper and the samples were washed three times with a drop of UPW. Subsequently, samples were dried in the stream of nitrogen and kept in a desiccator until further analysis.

#### 2.5.9. Atomic Force Microscopy (AFM)

For atomic force microscopy (AFM) imaging a drop of samples’ suspension (5 μL) was placed on hydrophilic freshly cleaved mica, attached to the metal disc. To remove unattached TiNMs the samples were washed with UPW after 10 min and left to dry in the air. AFM imagining in tapping mode under ambient conditions in the air was performed by a MultiMode probe atomic force microscope with a Nanoscope IIIa controller and a “J” scanner with a vertical engagement (JV) of 125 μm (Veeco Instruments, Bruker, Santa Barbara, CA, USA). A silicon tip (R-TESPA-525, Bruker, nom. freq. 525 kHz, nom. spring constant 100 N m^−1^) was used. The linear scanning rate was optimized between 1.0 and 1.5 Hz at the scan angle of 0°. For the analysis of the images the offline AFM NanoScope software (Bruker Corporation, Billerica, MA, USA), version 1.7, was used.

#### 2.5.10. Acellular Reactive Oxygen Species (ROS) Generation

To evaluate the ability of TiNMs and nanocomposites to induce ROS acellularly, the DCFH_2_-DA assay was employed. The DCFH_2_-DA dye (10 mmol L^−1^) was diluted to 1 mmol L^−1^ in methanol and subjected to alkaline hydrolysis by 0.01 mol L^−1^ NaOH for 30 min. Obtained DCFH_2_ was further diluted to 10 μmol L^−1^ with 10 mmol L^−1^ phosphate buffer saline (PBS) and used immediately (150 μL) by mixing with 150 μL of PBS dispersions of tested materials in a black 96-well plate. The final concentrations of each tested material were: 10, 100, and 500 mg L^−1^. The PBS solution was used as a negative control, while SIN-1 (concentration 1.25, 2.5, 5. and 10 mmol L^−1^) was used as a positive control. The fluorescence intensity of dye (*λ*_ex_ = 485 nm, *λ*_em_ = 530 nm) was recorded on a Victor^TM^ microplate reader (Perkin Elmer, Boston, MA, USA), immediately and in 30 min intervals. Between the measurements, the plate was kept at room temperature, protected from light.

To evaluate the possible interference of tested materials with DCFH_2_, the dye was dissolved in acetone to a concentration of 1 mmol L^−1^, hydrolyzed by 0.01 mol L^−1^ NaOH, and diluted to 50 μmol L^−1^ with PBS. The 150 μL of this solution was mixed with 150 μL of each material and the fluorescence (*λ*_ex_ = 485 nm, *λ*_em_ = 530 nm) was measured immediately. The same procedure was repeated with 10 mmol L^−1^ fluorescein prepared in PBS that was used as a blank in both interference experiments.

All experiments were performed in triplicate.

#### 2.5.11. Hemocompatibility Assay

Hemocompatibility experiments were conducted according to the modified Drabkin’s method. Whole blood (WB) samples were collected from six female healthy volunteers aged between 25 and 55 who were non-smokers, not pregnant, and free of medications for the previous 10 days. Written informed consent was obtained from each donor. Venous blood was collected into vacuum tubes (BD Vacutainer, Franklin Lakes, NJ, USA) containing potassium EDTA as an anticoagulant. The study protocol was performed following The Code of Ethics of the World Medical Association (Declaration of Helsinki) and approved by the Ethical Committee of University Hospital Osijek (approval number R2:18722–10/2015). The privacy rights of human subjects were observed and secured. The blood Hb levels were determined by adding 20 μL of whole blood to 5 mL of Drabkin’s reagent and measuring the absorbance at 540 nm. The Hb concentration was determined from the calibration curve prepared with Hb standard in PBS. Blood was then diluted with PBS to the Hb concentration of 10 ± 1 g L^−1^ and 100 μL of diluted blood was added to 900 μL of materials suspended in PBS. The final concentrations of tested materials were 10, 100, and 500 mg L^−1^. After incubation for 3 h at 37 °C, the mixtures were centrifuged at 800× *g* for 15 min to separate the erythrocytes. The supernatant was extracted and mixed with Drabkin’s reagent (1:1 *v*/*v*). The absorbance was measured at 540 nm using the Victor^TM^ microplate reader (Perkin Elmer, Waltham, MA, USA). The Hb concentrations in samples were calculated from the calibration curve. The percentage of hemolyzed erythrocytes was determined by dividing the measured Hb concentration in supernatants with the Hb concentration in diluted blood, corrected for dilution factors. The solution of 10 mg mL^−1^ polyethylene glycol was used as a negative control, while the positive control was 2% (*v*/*v*) Triton X-100. All measurements were conducted in triplicate.

Due to the possible false results emerging from the interference of materials with reagents [38], two additional experiments were conducted. The optical interference assay involved incubating the materials with diluted blood plasma (containing no Hb), mixing with Drabkin’s reagent, and measuring the absorbance. The blood plasma was prepared from the blood of the same donors and diluted with PBS in the same way as the blood in the hemolysis assay. The absorbance of materials containing mixtures and the one of the control containing PBS was compared to the absorbance of the control, containing PBS. The other possible interference arises from the tendency of proteins to bind to the nanomaterials’ (NMs) surface [39], thus decreasing the Hb from the supernatant. The adsorption of Hb to the NM surface was tested using 10 mg L^−1^ human Hb solution in diluted human plasma. The 100 μL of Hb solution was added to 900 μL of NM suspensions and the assay was completed following the same protocol as before. The Hb levels in mixtures containing NMs were compared to the control containing only PBS, and the recovery was calculated as a percentage of control.

#### 2.5.12. Data Analysis

To determine particle size distributions from SEM and TEM micrographs image analysis software Image J 1.48v (freely available at http://imagej.nih.gov/ij/, accessed on 15 March 2021) was used. For each sample, 50 particles were measured.

Crystallite sizes of CaDHA formed by precipitation in the presence of TiNMs were calculated using Scherrer Equitation (1) [40,41]:

(1) $$\mathrm{crystallite}\;\mathrm{size}=\frac{k\lambda }{\mathrm{FWHM}\;\cos\left(\theta \right)}$$

where *k* is the shape factor (*k* = 0.9), *λ* is the wavelength of Cu K_α_ radiation (*λ* = 0.154056 nm) FWHM is full width at half maximum of the peak at 2*θ* 32.0° (the reflection which in all investigated systems without ambiguity corresponds to CaDHA) and *θ* is diffraction angle.

The statistically significantly different ROS levels were determined by two-way analyses of variance (ANOVAs) SYSTAT Software, Inc., (San Jose, CA, USA; www.systatsoftware.com, accessed on 20 March 2021). The differences between the groups were established by Tukey and Scheffe’s post hoc test. The probability level was set at α = 0.05.

## 3. Results

### 3.1. TiNPls and TiNWs Characterization

**Figure 1:**
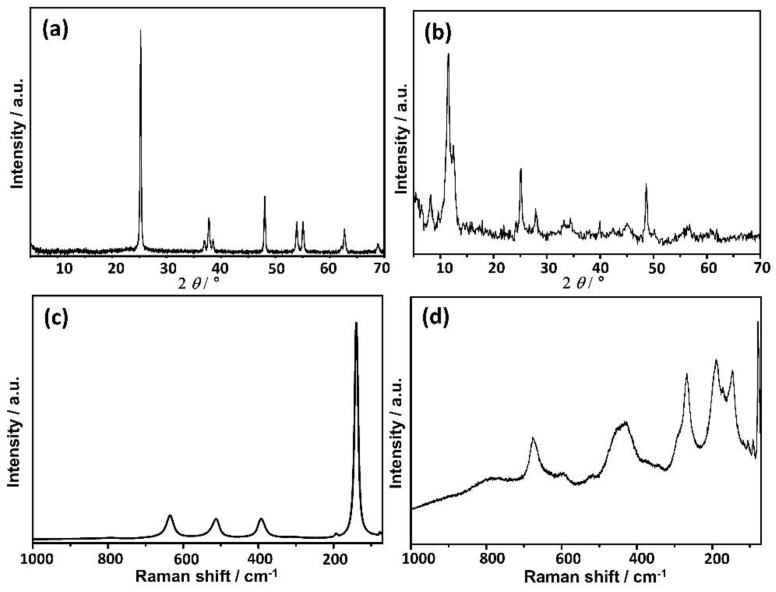
Powder X-ray diffraction (PXRD) patterns (**a**,**b**) and Raman spectra (**c**,**d**) of synthesized TiO_2_ nanoplates (**a**,**c**) and nanowires (**b**,**d**).

PXRD patterns and Raman spectra of synthesized TiNPls and TiNWs are given in Figure 1. PXRD pattern of TiNPls (Figure 1a) contained diffraction of anatase (JCPDS card 21-1272). Namely, peaks at 2*θ* 25.2°, 36.9°, 37.9°, 38.5°, 48.1°, 53.8°, 55.1°, 62.7°, and 68.8° corresponding to (101), (103), (004), (112), (200), (105), (211), (204) and (116) reflections were observed. Anatase has six allowed bands in the first-order Raman spectrum, namely A_1_g + 2 B_1_g + 3 Eg [42]. Bands detected at 140 and 635 cm^−1^ correspond to Eg, while the band at 394 cm^−1^ corresponds to B_1_g vibration. In addition, the band at 512 cm^−1^ corresponds to a doublet of A_1_g and B_1_g vibrations (Figure 1c).

Diffraction peaks detected in the PXRD pattern of TiNWs indicate the formation of the mixture of layered titanates and TiO_2_-B phase (Figure 1b) [34]. The low intensity peak at 2*θ* 8.18°, found at lower 2*θ* value than (001) reflection of trititanates [43], can be assigned to byproducts, as e.g., hydrated titanates [44]. The reflection observed at 2*θ* 11.53° is characteristic of layered titanate [44]. Peaks observed at higher 2*θ* values, namely at 25.06°, 27.90° and 48.58° are characteristic for (110), (002), and (020) reflections of TiO_2_(B) [45]. Raman spectrum (Figure 1d) is in good agreement with those previously published for TiNWs [34,45,46].

**Figure 2:**
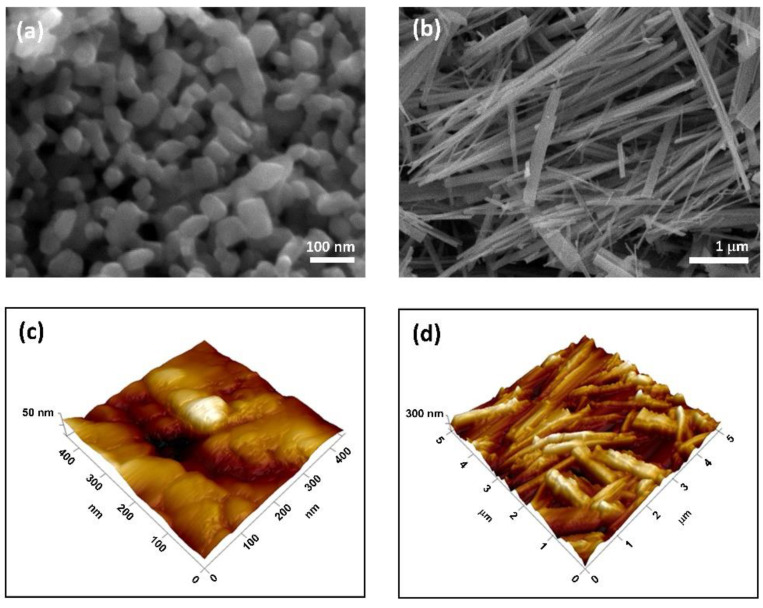
Scanning electron microscopy (SEM) (**a**,**b**) and atomic force microscopy (AFM) phases (**c**,**d**) micrographs of TiO_2_ nanoplates (**a**,**c**) and TiO_2_ nanowires (**b**,**d**).

SEM and AFM micrographs of TiNPls and TiNWs are shown in Figure 2. Micrographs (Figure 2a,c) confirm the formation of nanoplates of irregular cuboid shape, with an average size of 60.08 ± 18.35 nm similar to previous investigations [47,48]. The average length of synthesized TiNWs was 1677.17 ± 746.07 nm, and width 340.06 ± 112.50 nm as determined from SEM and TEM micrographs (Figure 2b,d).

The specific surface areas were 24.70 m^2^ g^−1^ and 21.49 m^2^ g^−1^ for TiNPls and TiNWs, respectively.

As NMs’ aggregation state is among important factors for mineralization, size distribution and zeta potential of TiNPls and TiNWs upon suspending in c-SBF and anionic reactant solution were determined (Table 1, Figure SI 1). The influence of the difference in media composition and ionic strength on the aggregation behavior of both TiNMs is clearly seen. In the anionic reactant solution, three populations of particles were detected in the suspension of both TiNMs, while in a c-SBF bimodal distribution of TiNPls and monomodal distribution of TINWs was observed. Both TiNPls and TiNWs have significantly more negative zeta potential in anionic reactant solution than in c-SBF.

### 3.2. Mineralization of TiNPls and TiNWs in Corrected Simulated Body Fluid (c-SBF)

From a physico-chemical perspective, biomineralization can be considered to be a precipitation of inorganic salts within an organic matrix. Biomimetic preparation of CaPs could, therefore, be simplified to their precipitation from media mimicking the composition of human plasma at 37 °C and atmospheric pressure. The artificial media most closely resembling the concentration of inorganic ions in human plasma is SBF [27,28]. SBF was primarily developed for the prediction of in vitro bioactivity of material by determining the ability of apatite formation on the material’s surface in SBF [49]. Soon it was realized that apatite formation in SBF can be used for biomimetic coating of different implant materials [14,27,50]. The advantage of such an approach is that, since it is a solution-based method, materials of different shapes and porosity can be coated. In addition, due to the mild conditions, the coprecipitation of biologically active molecules is also possible [51]. However, the procedure can be long lasting (up to 4 weeks) which was surpassed, in the case of titanium, by the pretreatment of the implant surface (acid, base, forming surface nanostructures) [14,52] or by increasing the concentrations of SBF (i.e., 1.5 × SBF, 5 × SBF) [29,53].

In this paper, the aim is to compare the immersion in c-SBF and spontaneous precipitation as two biomimetic (or close to it) procedures for coating TiNMs without any additional pretreatment. Therefore, the experiments were performed at 25 °C.

**Figure 3:**
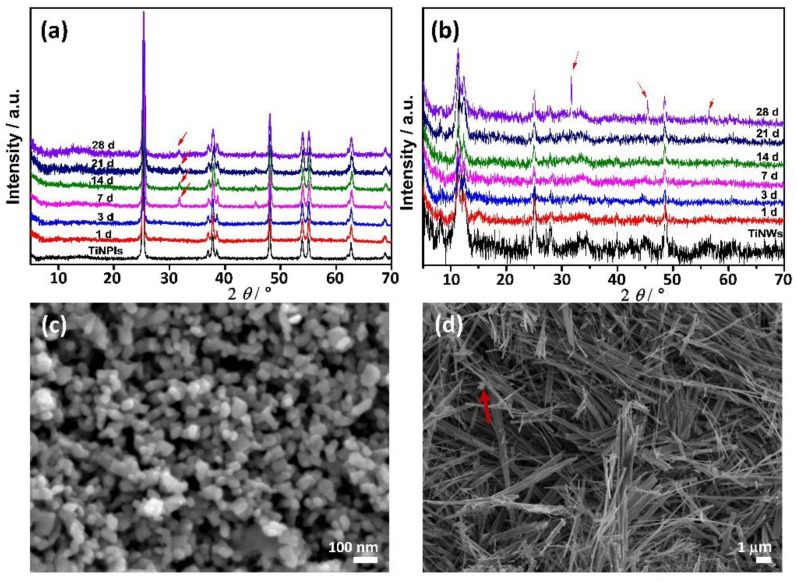
PXRD patterns (**a**,**b**) and SEM micrographs (**c**,**d**) of TiO_2_ nanoplates (TiNPls) (**a**,**c**) and TiO_2_ nanowires (TiNWs) (**b**,**d**) after soaking in corrected simulated body fluid for 1–28 d. SEM micrographs of the precipitates were obtained after 28 d. *ϑ* = (25.0 ± 0.1) °C. The red arrows indicate the presence of calcium deficient hydroxyapatite (CaDHA).

In Figure 3, PXRD patterns of TiNPls and TiNWs immersed for different periods of time in c-SBF, as well as SEM micrographs of the materials obtained after 28 d of immersion, are shown. The most intense reflection characteristic for apatites at 2*θ* 31.8° and 45.6° [54,55] were detected after 7 d of immersion of TiNPls. In the case of TiNWs, these reflections were observed only after 28 d. In the PXRD pattern of TiNWs after 28 d of immersion, the peak at 2*θ* 56.6° corresponding to apatites was also observed [55]. Spherical aggregates of small plate-like CaP’s crystals were sporadically observed in the case of TiNWs and not distinguishable in the case of TiNPls (Figure 3c,d).

Although TiNPls and TiNWs showed a low ability to induce CaP formation in SBF, as well as TiNPs and TiNTs investigated in a previous study [13], some trends in the influence of morphology and surface structure of TiNMs could be observed. The key step in CaPs’ formation on titanium is the presence of Ti-OH groups on the surface that interact with positively charged calcium ions forming calcium titanate [56]. A positively charged surface attracts phosphate ions and amorphous CaP is formed, which further transforms to a more stable crystalline apatite. At first glance, this would mean that the induction ability of the nanomaterial would be greater if its zeta potential is more negative. However, CaP is formed earlier on TiNPls, TiNPs, and TiNTs than on TiNWs which in c-SBF had the most negative zeta potential among investigated TiNMs [13]. This indicates that, as shown by Uchida et.al., a specific arrangement of Ti-OH groups is also needed and it depends both on NMs composition and morphology [57]. TiNPls used in this work, and TiNPs from our recent study [13] are both anatase. However, CaP formation on TiNPs was observed already after the first day of soaking which can be attributed to the different morphology.

### 3.3. Mineralization of TiNPls and TiNWs by Spontaneous Precipitation 

Another possible route of biomimetically coating TiNMs is to use them as a suspended substrate during CaP’s precipitation at physiological pH. In our recent article, we have shown that by using precipitation systems with higher supersaturation than in c-SBF it is possible to coat TiNPs and TiNTs with CaDHA [13]. To the best of our knowledge, no other such attempts were made by other groups. The closest to this procedure were Enayati-Jazi et al. [17] who prepared HAP/TiO_2_ nanorod composites by in situ precipitation at 70 °C.

**Figure 4:**
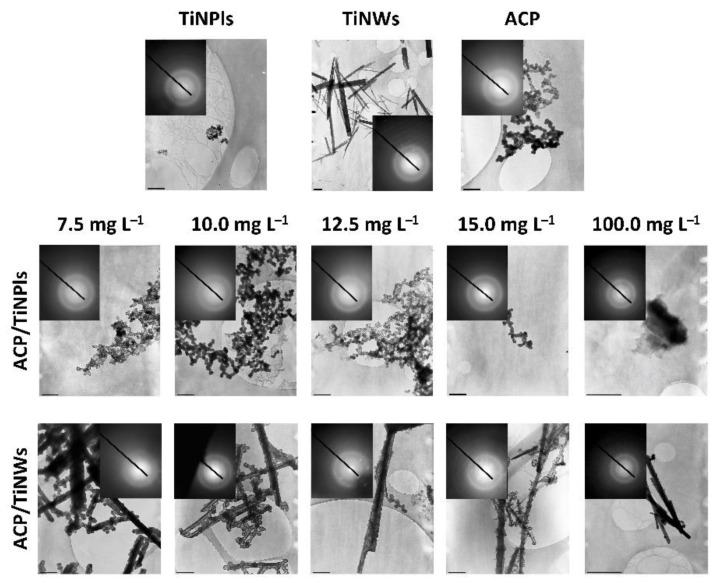
TEM micrographs of the TiO_2_ nanoplates (TiNPls), nanowires (TiNWs), amorphous calcium phosphate (ACP) formed in the control system, and a precipitate formed in the presence of a different concentration of TiNPls (ACP/TiNPls) and TiNWs (ACP/TiNWs) after 15 min reaction time. *c* (CaCl_2_) = *c* (Na_2_HPO_4_) = 4 · 10^−3^ mol dm^−3^, pH_init_ = 7.4, *ϑ* = (25.0 ± 0.1) °C. The scale bar is 0.5 μm, except in 100 mg L^−1^ cases where the scale is 1 μm.

**Figure 5:**
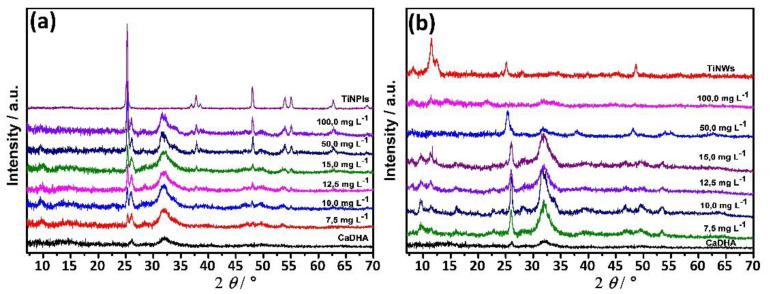
PXRD patterns of the precipitate formed in the control system (calcium-deficient hydroxyapatite, CaDHA) and in the presence of (**a**) TiO_2_ nanoplates (TiNPls) and (**b**) TiO_2_ nanowires (TiNWs) after 60 min reaction time. *c* (CaCl_2_) = *c* (Na_2_HPO_4_) = 4 · 10^−3^ mol dm^−3^, pH_init_ = 7.4, *ϑ* = (25 ± 0.1) °C.

**Figure 6:**
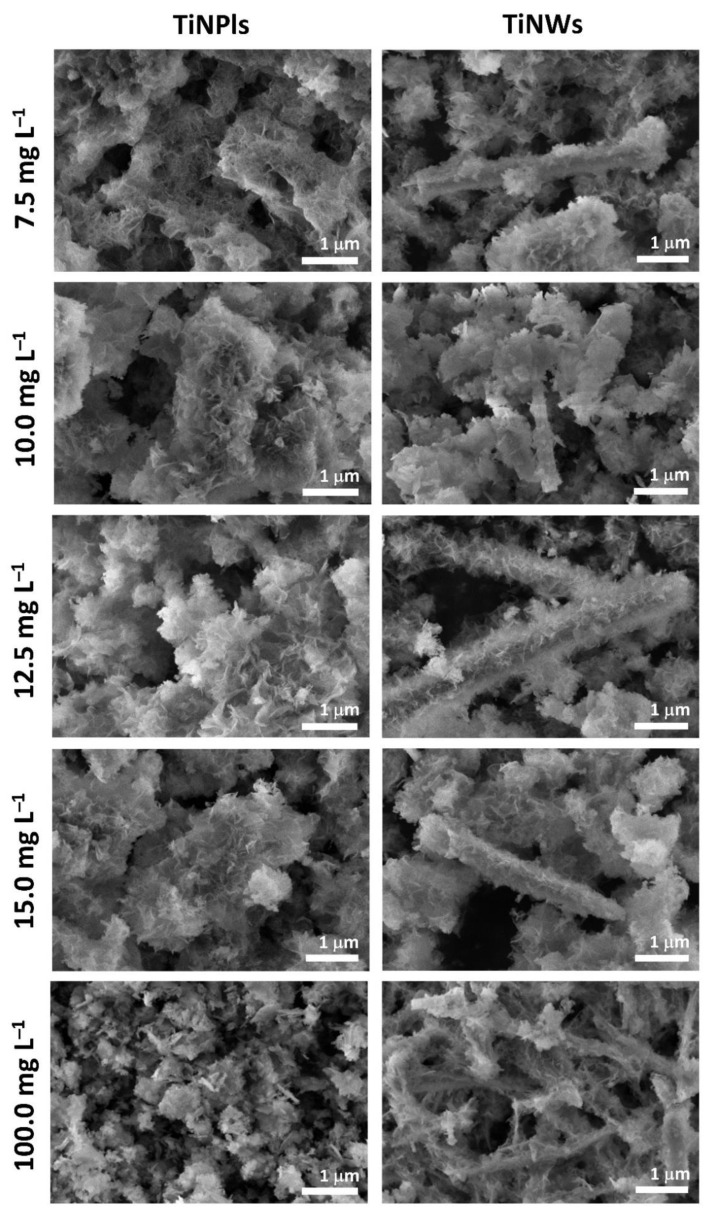
SEM micrographs of the precipitate formed in the presence of different concentration of Table 2. nanoplates (TiNPls) and nanowires (TiNWs) after 60 min reaction time. *c* (CaCl_2_) = *c* (Na_2_HPO_4_) = 4 · 10^−3^ mol dm^−3^, pH_init_ = 7.4, *ϑ* = (25 ± 0.1) °C.

From neutral and basic solutions CaPs usually precipitate in two steps [58]. In the first step, an amorphous precursor (amorphous calcium phosphate, ACP) is formed. In contact with the mother liquor, ACP may transform in OCP, DCPD, and/or HAP, depending on experimental conditions [59,60,61]. In all investigated precipitation systems, CaP is formed by this pathway, as confirmed by pH vs. time curves (Figure SI 2, Table SI 1), TEM and SEM micrographs, and PXRD patterns (Figure 4, Figure 5 and Figure 6).

TEM micrographs and selected area electron diffraction (SAED) patterns of the precipitates formed after 15 min aging time revealed that ACP is the first phase formed in the presence and absence of TiNMs (Figure 6). Typical chain-like aggregates of spherical ACP particles [59,61,62] are observed in all investigated systems. TiNPls are incorporated in chain-like aggregates of spherical ACP particles. Already from the lowest investigated TiNWs’ concentrations, ACP particles are formed on the surface of nanowires. As well some individual ACP particles, not formed on the surface of TiNWs, were detected. The size of ACP particles formed on the surface increases with the TiNWs’ concentration, from 83.8 ± 23.0 nm at 7.5 mg L^−1^ TiNWs to 123.5 ± 55 nm at 100.0 mg L^−1^. This indicates that the surface of TiNWs can be an efficient substrate for the growth of ACP, probably because of the large negative zeta potential that they have in the precipitation system.

PXRD patterns of the precipitates formed after 60 min aging time confirm that ACP has transformed into a crystalline phase (Figure 5). In the absence of TiNMs, two prominent reflections at 2*θ* around 26.0° and 32.0° as well as low intensity reflections at 2*θ* around 46.5°, 50.0° and 53.5° characteristic of CaDHA [54,55] were observed. Same reflections were observed in PXRD patterns of precipitates formed in the different concentrations of TiNMs, indicating that neither TiNM induces a change in precipitate composition. As expected, the intensity of characteristic TiNPls’ reflections increased with TiNPls’ concentration. It is worth noting that, as in the case of TiNPls and TiNWs, in the presence of TiNPs and TiNTs only CaDHA was formed [13], indicating that in applied conditions neither structure nor morphology of TiNMs influences the composition of formed CaPs. However, the presence of TiNMs influenced the crystalline size (Table 2). At TiNMs concentrations of up to 15 mg L^−1^ the crystalline sizes have similar values to those obtained in the control system. At the 50 mg L^−1^ concentration, the crystalline sizes increased in the presence of both TiNMs. At the highest investigated TiNMs concentration, in the presence of TiNPls crystallite size was still larger than in the control system, while in the presence of TiNWs it was significantly reduced.

SEM micrographs of the formed CaDHA/TiNM composites are shown in Figure 6. In the absence of TiNMs, CaDHA precipitates in the form of typical irregular aggregates of thin leaf-like crystals (Figure SI 3) [63]. In the presence of an increasing concentration of TiNPls, the crystals are progressively more formed on the surface of the platelets, as confirmed by the more cuboid appearance of the aggregates. A significant change in morphology was observed at the highest applied TiNPls’ concentration (100 mg L^−1^). Smaller aggregates and crystals are formed and a small amount of somewhat thicker plate-like crystals are observed. No uncoated TiNPls were observed. In the presence of TiNWs at lower concentrations, along with TiNWs coated with CaDHA, aggregates of CaDHA leaf-like crystals not formed on TiNWs were observed. As expected, the amount of CaDHA which was not formed on TiNWs decreased with TiNWs’ concentration. At the highest investigated concentration all CaDHA formed on the surface of TiNWs as an irregular layer of leaf-like crystals. Comparing the results from all four investigated TiNMs, a similar influence on morphology was observed for TiNPls and TiNPs on the one hand [13], and TiNTs and TiNWs on the other. This indicates an important effect of the NMs’ morphology.

Additional physico-chemical and biological characterization was performed on the composites formed in the presence of 100 mg L^−1^ TiNMs to assess the potential of CaDHA/TiNMs composites for biomedical application. BET surface areas of the TiNMs and composites are given in Table 3. For both composite materials, the surface areas are larger than for TiNMs but smaller than for CaDHA.

**Figure 7:**
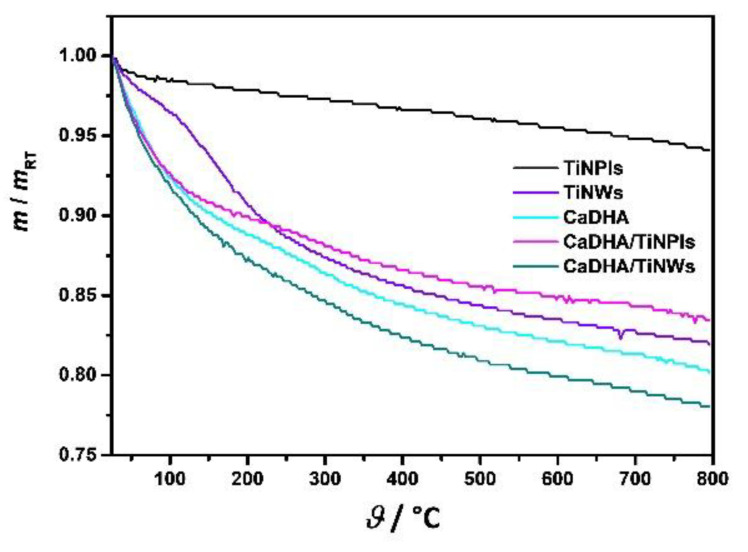
Thermogravimetric analysis (TGA) curves of the TiO_2_ nanoplates (TiNPls) and nanowires (TiNWs), precipitates formed after 60 min reaction time in the control system (calcium deficient hydroxyapatite, CaDHA [13]) and in the presence of 100 mg L^−1^ TiO_2_ nanoplates (CaDHA/TiNPls) and nanowires (CaDHA/TiNWs). *c* (CaCl_2_) = *c* (Na_2_HPO_4_) = 4 · 10^−3^ mol dm^−3^, pH_init_ = 7.4, *ϑ* = (25 ± 0.1) °C.

TGA curve of TiNPls showed a two-step loss of weight (Figure 7). Initial abrupt loss of weight of 1.3% at temperatures up to 80 °C corresponds to the loss of water, while subsequent continuous weight loss of 3.4% can be attributed to the loss of water adsorbed between parallel TiNPls and dehydroxylation according to Equation (2) [48]:

TiO_2−*x*_(OH)_2*x*_ → TiO_2_ + *x*H_2_O (2)

On the other hand, the TGA curve of TiNWs shows three different mass losses. At temperatures up to 150 °C, around 4% weight loss is detected, while in the temperature range 105–250 °C an additional 7% of the mass was lost. Furthermore, 7% of the mass is lost by heating up to 800 °C. The thermal decomposition of CaDHA proceeds in three steps. The weight loss of 9% at temperatures up to 126 °C, can be attributed to the loss of absorbed water. The CaDHA decomposition at temperatures above 126 °C proceeds in two steps, totaling a weight loss of 11% which is in agreement with the literature data [64,65]. The TGA curves of composites have a similar shape as the curve of CaDHA. However, these samples differ in total weight loss. The largest weight loss was observed for CaDHA/TiNWs composite.

**Figure 8:**
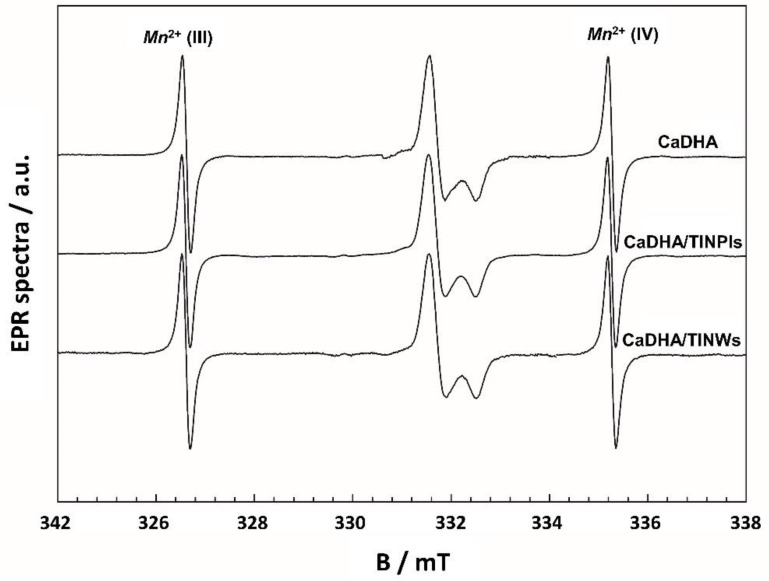
Experimental EPR spectra of gamma-irradiated precipitates formed after 60 min reaction time in the control system (calcium deficient hydroxyapatite, CaDHA) and the presence of 100 mg L^−1^ TiO_2_ nanoplates (CaDHA/TiNPls), and nanowires (CaDHA/TiNWs). Two satellite lines on the left and right side are the third and fourth lines of the Mn^2+^/MgO internal standard.

EPR spectra and corresponding spectroscopic parameters are a reflection of the microstructure of composites and their constituents. To verify those changes, EPR spectroscopy was used to monitor the quality of synthesis and impact of sterilization by irradiation. No observable EPR spectra were detected for the non-irradiated samples confirming that no traces or defects of possible metal ions or residual radicals from the synthesis are present (not shown). As expected, after the sterilization process, the impact of gamma radiation has been observed and radiation-induced radicals were simultaneously used to monitor the structural changes regarding the type of sample. In Figure 8, experimental EPR spectra of the control system (CaDHA), CaDHA/TiNPls, and CaDHA/TiNWs are shown. In line with our previous investigations [13], the room temperature EPR spectrum of the control system is composed of an axially symmetric $CO_{2}^{-}$ radical (*g_⊥_* = 2.0025 and *g_II_* = 1.9972). All experimentally obtained spectra can be simulated on the same spectroscopic parameters (Figure SI 4 and Table SI 2). EPR spectra are formed by contributions of the same type of paramagnetic center as confirmed by the same shape of the spectra. Thus, it can be concluded that the composites with the addition of the TiNPls and TiNWs do not create changes in the local structure of CaDHA.

### 3.4. Acellular ROS Generation

Induction of ROS generation by biomaterials could play both beneficial and non-beneficial roles. Increased ROS generation might be used in different therapies, like cancer treatment [66], drug delivery [67], or antimicrobial application [68]. However, if ROS generation and elimination are not balanced, elevated levels of ROS may damage DNA, proteins, and cells [67]. A number of studies have shown that increased generation of ROS weakens the osteoblast functions and new bone formation [69,70] pointing that generation of the ROS at the place of implantation would negatively affect the healing process [71]. This motivates the search for strategies for preventing or reducing ROS formation at the bone/biomaterial interface [72]. It is well known that TiNMs are able to generate ROS under ultraviolet (UV) irradiation [45,66,73], but also non-photocatalitically [74]. CaPs also can induce elevated ROS levels. Recent investigation has shown that treatment with biphasic CaPs prepared by solution combustion synthesis can increase intracellular ROS generation by almost 60% as compared to non-treated cells [75]. It was also shown that the HAP nanoparticles can cause a gradual increment in the intracellular ROS levels in a dose-dependent manner [76] and that their effect also depends on particles’ size [77] and morphology [78].

**Figure 9:**
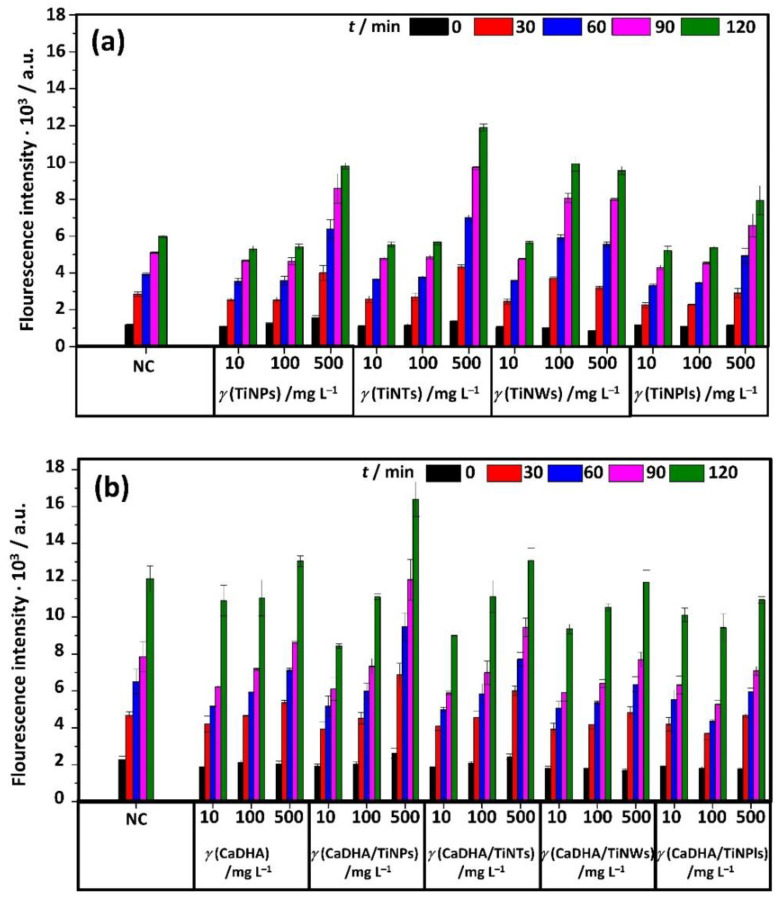
The intensity of DCF fluorescence after incubation in PBS with different concentrations (10, 100, and 500 mg L^−1^) of (**a**) TiO_2_ nanoparticles (TiNPs), titanate nanotubes (TiNTs), TiO_2_ nanowires (TiNWs), and TiO_2_ nanoplates (TiNPls), and (**b**) precipitates formed after 60 min reaction time in the control system (calcium deficient hydroxyapatite, CaDHA) and in the presence of 100 mg L^−1^ TiO_2_ nanoparticles (CaDHA/TiNPs), nanotubes (CaDHA/TiNTs), nanowires (CaDHA/TiNWs), and nanoplates (CaDHA/TiNPls).

Therefore, the ability of CaDHA/TiNMs composites for acellular ROS generation was determined in order to evaluate their safety towards biomedical application. The results of acellular ROS assay are given in Figure 9. Both TiNMs (Figure 9a) and CaDHA/TiNMs composites (Figure 9b) showed dose-dependent behavior. Similar dose-dependent behavior of TiNM with different morphologies (i.e., particles, tubes, and cubes) and quartz NPs was previously observed [79]. In Figure 9, the negative control (NC) represents the situation with no ROS generation, and the fluorescence increase in time is only due to DCFH_2_ oxidation in the air. The positive control (PC) shows the assay to be functioning properly, as the dose- and time-dependent increase in fluorescence intensity is noted (Figure SI 5). Possible interferences, namely fluorescence quenching by NMs and NMs’ autofluorescence, were found to be not significant (Table SI 3). All tested materials generate smaller or not statistically different ROS levels compared to the NC when applied in concentrations 10 and 100 mg L^−1^ (Figure 9). The only exception are TiNWs for which a significantly higher ROS level was observed at concentration 100 mg L^−1^ from 30 min incubation time. There are several possible explanations for ROS levels lower than NC observed for some materials. Fluorescence quenching is one of them, but the test of interference has shown that it is well below significant levels (Table SI 3). Another reason may be the antioxidative activity of composites. Indeed, the antioxidant activity of some NMs and composites at low doses was previously evidenced [80,81]. As well interference through mechanisms other than fluorescence quenching, such as binding and precipitation of the dye [38] may be the cause. This may only be visible at lower material concentrations since the pro-oxidant effect at higher doses may counteract it. The TiNPs and TiNTs induced significant ROS at the highest tested concentration as compared to NC, already from the earliest incubation time. However, in the case of composites, elevated ROS levels were only observed for CaDHA/TiNPs composites. Even in this case, the amount of generated ROS was lower compared to NC in the presence of CaDHA/TiNPs composite than in the presence of TiNPs. This indicates that the CaDHA/TiNMs composites generate a lower amount of ROS as compared to TiNMs and CaDHA alone. Therefore, the tested composites can be considered ROS-inactive in the acellular system.

### 3.5. Hemocompatibility of TiNMs and CaP/TiNMs Composites

**Figure 10:**
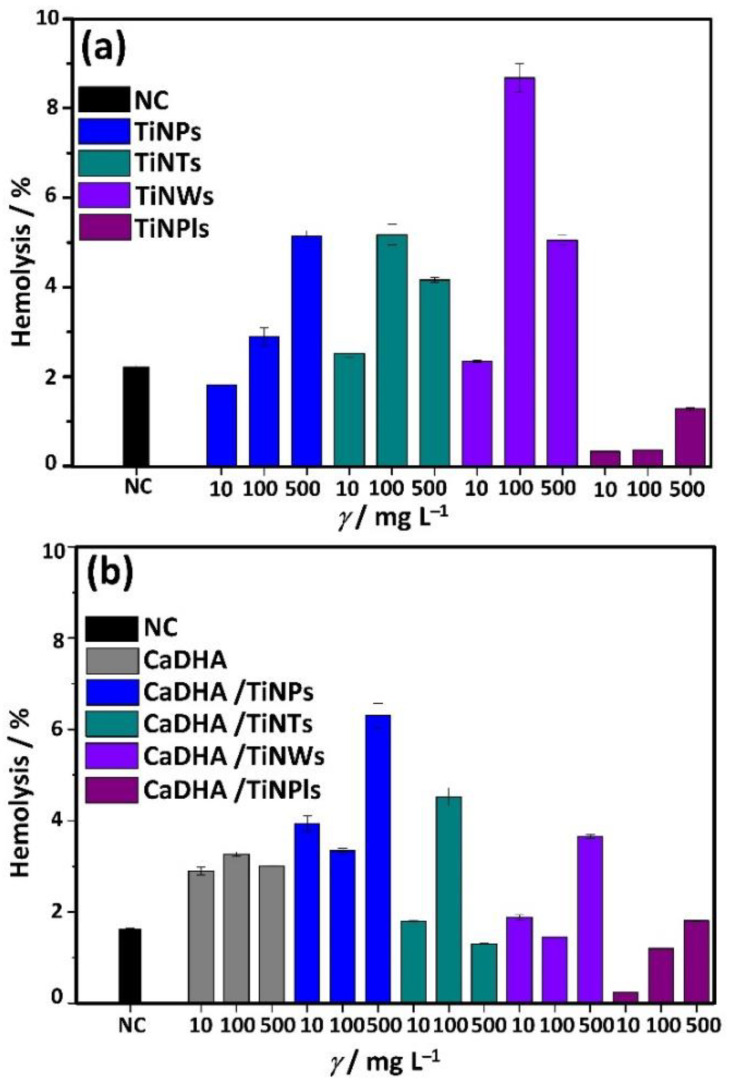
The results of the hemolysis assay on different concentrations (10, 100, and 500 mg L^−1^) of (**a**) TiO_2_ nanoparticles (TiNPs), titanate nanotubes (TiNTs), TiO_2_ nanowires (TiNWs), and TiO_2_ nanoplates (TiNPls), and (**b**) precipitates formed after 60 min of reaction time in the control system (calcium deficient hydroxyapatite, CaDHA) and the presence of 100 mg L^−1^ TiO_2_ nanoparticles (CaDHA/TiNPs), nanotubes (CaDHA/TiNTs), nanowires (CaDHA/TiNWs), and nanoplates (CaDHA/TiNPls) suspended in phosphate buffered saline (PBS).

Hemocompatibility is among the major criteria for approving the usage of any biomaterial as a medical device. Therefore, adverse interactions between new materials and blood should be analyzed [82]. In this study, hemocompatibility was determined by a modified Drabkin’s method that determines the hemolytic action of a tested agent. The hemolysis of erythrocytes incubated with TiNMs and CaDHA/TiNMs composites was expressed as the percentage of Hb released from erythrocytes after the incubation with tested materials relative to the original Hb concentration (Figure 10). Optical interferences of tested TiNMs and CaDHA/TiNMs composites in the absence of Hb as well as interferences that may arise from Hb adsorption on materials could be excluded (Table SI 4).

According to the F756-08 standard of the American Society for Testing and Materials (Standard Practice for Assessment of Hemolytic Properties of Materials; 2008), the material is considered non-hemolytic if the percentage of the hemolysis is in the range of 0–2%, slightly hemolytic if the range is 2–5%, and hemolytic if the percentage of hemolysis is above 5%. However, scientific literature usually considers that a material is hemocompatible if the hemolysis percentage is less than 10%, while below 5% the material is considered highly hemocompatible [83,84,85].

All tested materials showed a hemolysis percentage lower than 10% in the case of concentrations lower than 100 mg L^−1^ (Figure 9). The percentage of hemolysis higher than 5% was determined for the highest investigated concentration of TiNPs, TiNTs, and TiNWs. Similar results were reported earlier for TiNPs and TiNWs [19] or Ti coated with a TiO_2_ nanotubes layer [86]. Among composites, only CaDHA/TiNT composites showed hemolysis higher than 5% at the highest concentration applied. This indicates that the compatibility of TiNMs were improved in CaDHA/TiNM, which is in agreement with literature data that have shown that nanosized and/or nanoporous HAP is a highly hemocompatible material [84,87,88].

## 4. Conclusions

In this work, the precipitation of CaP at room temperature in the presence of TiNPls and TiNWs as suspended substrates was investigated to verify its applicability as a fast and versatile method for preparing calcium phosphate composites with TiO_2_ nanomaterials of different morphologies and composition. CaDHA composites with both TiNMs were formed within 1 h of reaction time, contrary to immersion in c-SBF, where even after 28 d a significant amount of CaP had not been formed. At the employed experimental conditions, the precipitation process proceeded through two steps. First was the formation of ACP, which subsequently transformed in CaDHA. Due to their smaller size, TiNPls were embedded within chain-like aggregates of spherical ACP particles, while ACP particles were formed on the surface of TiNWs. CaDHA was the only CaP phase formed in the precipitation system without TiNMs addition. This indicates that TiNMs do not influence the CaP’s composition, indicating that the type of CaP’s phase formed could be tailored by adjusting conditions in the precipitation system such that the formation of a certain CaP phase is favored. Another advantage of the proposed preparation procedure, based on precipitation, in addition to being much faster, is the simpler media containing only sources of calcium and phosphate ions, unlike SBF which mimics the concentration of inorganic ions in human plasma. As SBF is a metastable solution, the problems of precipitation during its preparation are frequent and it should be stored for 1 month before usage [28]. Therefore, it can be concluded that the tested procedure based on spontaneous precipitation is also economically more acceptable.

In addition, CaDHA/TiNMs composites generate low levels of ROS and were hemocompatible, confirming their high potential in biomedical applications.

## Figures and Tables

**Table 1:** Hydrodynamic diameter (*d*_h_) and zeta potential (*ζ*) of TiO_2_ nanoplates (TiNPls) and nanowires (TiNWs) suspended in corrected simulated body fluid (c-SBF) and anionic reactant solution (*c* (Na_2_HPO_4_) = 8 · 10^−3^ mol dm^−3^, pH_init_ = 7.4), *ϑ* = (25.0 ± 0.1) °C.

	TiNPls			TiNWs		
	*d*_h_/nm	vol. %	*ζ*/mV	*d*_h_/nm	vol. %	*ζ*/mV
c-SBF	847.8 ± 32.0	92.0 ± 5.2	−10.5 ± 0.1	1078.0 ± 56.3	100	−8.94 ± 0.6
	5357.8 ± 137.8	8.0 ± 5.2				
Anionic Reactant Solution	216.2 ± 3.5	57.0 ± 14.9	−53.0 ± 2.5	235.5 ± 32.1	1.4 ± 2.1	−55.0 ± 2.0
	1076.1 ± 170.7	31.1 ± 14.6		874.8 ± 34.3	93.3 ± 2.4	
	4743.7 ± 343.4	11.9 ± 2.4		5496.0 ± 100.0	5.3 ± 3.6	

**Table 2:** Crystallite size of calcium-deficient hydroxyapatite (CaDHA) formed in the control system and in the presence of TiO_2_ nanoplates (TiNPls) and titanate nanowires (TiNWs) after 60 min reaction time. *c* (CaCl_2_) = *c* (Na_2_HPO_4_) = 4 · 10^−3^ mol dm^−3^, pH_init_ = 7.4, *ϑ* = (25 ± 0.1) °C.

*γ*/mg L^−1^	Crystallite Size/nm	
	TiNPls	TiNWs
0	3.92	
7.5	3.97	4.01
10.0	3.71	4.89
12.5	3.74	3.85
15.0	3.48	3.72
50.0	5.28	4.36
100.0	4.89	1.94

**Table 3:** Brunauer–Emmett–Teller (BET) surface area of the TiO_2_ nanoplates (TiNPls), nanowires (TiNWs), precipitate formed after 60 min reaction time in the control system (calcium deficient hydroxyapatite, CaDHA) and in the presence of 100 mg L^−1^ TiO_2_ nanoplates (CaDHA/TiNPls) and nanowires (CaDHA/TiNWs). *c* (CaCl_2_) = *c* (Na_2_HPO_4_) = 4 · 10^−3^ mol dm^−3^, pH_init_ = 7.4, *ϑ* = (25 ± 0.1) °C.

Material	S_BET_/m^2^ g^−1^
TiNPls	24.70
TiNWs	21.49
CaDHA	81.81
CaDHA/TiNPls	51.66
CaDHA/TiNWs	35.80

## Data Availability

The data are available within the article or its supplementary materials.

## Supplementary Materials

The following are available online at https://www.mdpi.com/article/10.3390/nano11061523/s1, Figure SI 1: Volume size distributions of TiNPls (a,b) and TiNWs (c,d) in c-SBF (a,c) and anionic reactant solution (*c* (Na_2_HPO_4_) = 8 · 10^−3^ mol dm^−3^, pH_init_ = 7.4, *ϑ* = (25.0 ± 0.1) °C, Figure SI 2: Representative pH vs. time curves obtained in the control system (without TiO_2_ nanomaterials) and in the presence of different concentrations of TiO_2_ (a) nanoplates (TiNPls) and (b) nanowires (TiNWs). *c* (CaCl2) = *c* (Na_2_HPO_4_) = 4 · 10^−3^ mol dm^−3^, pH_init_ = 7.4, *ϑ* = (25 ± 0,1) °C, Table SI 1: Average induction time obtained from pH vs. time curves from 3 measurements with standard deviations in the control system (without TiO_2_ nanomaterials) and in the presence of different concentrations of TiO_2_ nanoplates (TiNPls) and nanowires (TiNWs). *c* (CaCl_2_) = *c* (Na_2_HPO_4_) = 4 · 10^−3^ mol dm^−3^, pH_init_ = 7.4, *ϑ* = (25.0 ± 0.1) °C, Figure SI 3: SEM micrographs of the precipitate formed in control system (calcium deficient hydroxyapatite, CaDHA) after 60 min reaction time. *c* (CaCl_2_) = *c* (Na_2_HPO_4_) = 4 · 10^−3^ mol dm^−3^, pH_init_ = 7.4, *ϑ* = (25 ± 0.1) °C, Figure SI 4: Simulated (red) and experimental (dark) EPR spectra of gamma irradiated precipitates formed after 60 min reaction time in control system (calcium deficient hydroxyapatite, CaDHA) and in the presence of 100 mg L^−1^ TiO_2_ nanoplates (CaDHA/TiNPls), and nanowires (CaDHA/TiNWs), Table SI 2: EPR spectral parameters extracted by simulation of experimental spectra according to Figure 8 and Figure SI 4 for precipitates formed after 60 min reaction time in control system (calcium deficient hydroxyapatite, CaDHA) and in the presence of 100 mg L^−1^ TiO_2_ nanoplates (CaDHA/TiNPls), and nanowires (CaDHA/TiNWs), Figure SI 5: The intensity of DCF fluorescence after incubation in PBS with different concentrations of SIN-1 (NC—negative control; PC—positive control) used for comparison with (a) TiO_2_ nanoparticles (TiNPs), titanate nanotubes (TiNTs), TiO_2_ nanowires (TiNWs), and TiO_2_ nanoplates (TiNPls), and (b) precipitates formed after 60 min reaction time in control system (calcium deficient hydroxyapatite, CaDHA) and in the presence of 100 mg L^−1^ TiO_2_ nanoparticles (CaDHA/TiNPs), nanotubes (CaDHA/TiNTs), nanowires (CaDHA/TiNWs), and nanoplates (CaDHA/TiNPls), Table SI 3: The % of fluorescence quenching and autofluorescence of different concentrations of TiO_2_ nano-particles (TiNPs), titanate nanotubes (TiNTs), TiO_2_ nanowires (TiNWs), TiO_2_ nanoplates (TiNPls), and precipitates formed after 60 min reaction time in control system (calcium deficient hydroxyapatite, CaDHA) and in the presence of 100 mg L^−1^ TiO_2_ nanoparticles (CaDHA/TiNPs), nanotubes (CaDHA/TiNTs), nanowires (CaDHA/TiNWs), and nanoplates (CaDHA/TiNPls), Table SI 4: The results of optical interference assay and adsorption assay on different concentrations of TiO_2_ nanoparticles (TiNPs), titanate nanotubes (TiNTs), TiO_2_ nanowires (TiNWs), TiO_2_ nanoplates (TiNPls), and precipitates formed after 60 min of reaction time in control system (calcium deficient hydroxyapatite, CaDHA) and in the presence of 100 mg L^−1^ TiO_2_ nanoparticles (CaDHA/TiNPs), nanotubes (CaDHA/TiNTs), nanowires (CaDHA/TiNWs), and nanoplates (CaDHA/TiNPls) in hemolysis test.
