# Supplementary material for: Precipitation at Room Temperature as a Fast and Versatile Method for Calcium Phosphate/TiO2 Nanocomposites Synthesis
Source: Nanomaterials (Basel). 2021 Jun 9;11(6):1523. doi: 10.3390/nano11061523 (PMC8230063; doi:10.3390/nano11061523)
Supplement: Supplementary file 1 [file nanomaterials-11-01523-s001.zip › nanomaterials-1210209-supplementary.pdf]

nanomaterials

MDPI

Supplementary information for

Precipitation at Room Temperature as a Fast and Versatile Method for Calcium Phosphate/TiO_2 Nanocomposites Synthesis

Ina Erceg [1], Atiđa Selmani [1], Andreja Gajović [2], Borna Radatović [3], Suzana Šegota [1], Marija Ćurlin [4], Vida Strasser [1], Jasminka Kontrec [5], Damir Kralj [5], Nadica Maltar-Strmečki [6], Rinea Barbir [7], Barbara Pem [7], Ivana Vinković Vrček [7] and Maja Dutour Sikirić [1,*]

1 Laboratory for Biocolloids and Surface Chemistry, Ruđer Bošković Institute, Bijenička Cesta 54, 10 000 Zagreb, Croatia; ierceg@irb.hr (I.E.); aselmani@irb.hr (A.S.); ssegota@irb.hr (S.Š.); Vida.Strasser@irb.hr (V.S.)
2 Laboratory for Energy Conversion Materials and Sensors, Ruđer Bošković Institute, Bijenička Cesta 54, 10000 Zagreb, Croatia; gajovic@irb.hr
3 Institute of Physics, Bijenička Cesta 46, 10000 Zagreb, Croatia; bradatovic@ifs.hr
4 Department of Histology and Embryology, School of Medicine, University of Zagreb, Šalata 3, 10000 Zagreb, Croatia; marija.curlin@mef.hr
5 Laboratory for Precipitation Processes, Ruđer Bošković Institute, Bijenička Cesta 54, 10000 Zagreb, Croatia; lugaric@irb.hr (J.K.); kralj@irb.hr (D.K.)
6 Laboratory for Magnetic Resonances, Ruđer Bošković Institute, Bijenička cesta 54, 10000 Zagreb, Croatia; nstrm@irb.hr
7 Institute for Medical Research and Occupational Health, Ksaverska Cesta 2, Zagreb, 10000, Croatia; rbarbir@imi.hr (R.B.); bpem@imi.hr (B.P.); ivinkovic@imi.hr (I.V.V.)
* Correspondence: sikiric@irb.hr; Tel.: +385-1-456-0941

Content of listed material:

Figure SI 1. Volume size distributions of TiNPls (a,b) and TiNWs (c,d) in c-SBF (a,c) and anionic reactant solution (c (Na_2HPO_4) = $8 \cdot 10^{-3}$ mol dm^{-3}, pH$_{init}$ = 7.4, ϑ = (25.0 ± 0.1) °C.

Figure SI 2. Representative pH vs time curves obtained in the control system (without TiO_2 nanomaterials) and in the presence of different concentrations of TiO_2 a) nanoplates (TiNPls) and b) nanowires (TiNWs). c (CaCl2) = c (Na_2HPO_4) = $4 \cdot 10^{-3}$ mol dm^{-3}, pH$_{init}$ = 7.4, ϑ = (25 ± 0,1) °C.

Table SI 1. Average induction time obtained from pH vs time curves from 3 measurements with standard deviations in the control system (without TiO_2 nanomaterials) and in the presence of different concentrations of TiO_2 nanoplates (TiNPls) and nanowires (TiNWs). c ($CaCl_2$) = c (Na_2HPO_4) = $4 \cdot 10^{-3}$ mol dm^{-3}, pH$_{init}$ = 7.4, ϑ = (25.0 ± 0.1) °C.

Figure SI 3. SEM micrographs of the calcium deficient hydroxyapatite (CaDHA) formed in control system after 60 min reaction time. c ($CaCl_2$) = c (Na_2HPO_4) = $4 \cdot 10^{-3}$ mol dm^{-3}, pH$_{init}$ = 7.4, ϑ = (25 ± 0.1) °C.

Figure SI 4. Simulated (red) and experimental (dark) EPR spectra of gamma irradiated precipitates formed after 60 min reaction time in control system (calcium deficient hydroxyapatite, CaDHA) and in the presence of 100 mg L^{-1} TiO_2 nanoplates (CaDHA/TiNPls), and nanowires (CaDHA/TiNWs).

Table SI 2. EPR spectral parameters extracted by simulation of experimental spectra according to Figures 8 and SI 4 for precipitates formed after 60 min reaction time in control system (calcium deficient hydroxyapatite, CaDHA) and in the presence of 100 mg L^{-1} TiO_2 nanoplates (CaDHA/TiNPls), and nanowires (CaDHA/TiNWs).

Figure SI 5. The intensity of DCF fluorescence after incubation in PBS with different concentrations of SIN-1 (NC – negative control; PC – positive control) used for comparison with a) TiO_2 nanoparticles (TiNPs), titanate nanotubes (TiNTs), TiO_2 nanowires (TiNWs), and TiO_2 nanoplates (TiNPls), and b) precipitates formed after 60 min reaction time in control system (calcium deficient hydroxyapatite, CaDHA) and in the presence of 100 mg L^{-1} TiO_2 nanoparticles (CaDHA/TiNPs), nanotubes (CaDHA/TiNTs), nanowires (CaDHA/TiNWs), and nanoplates (CaDHA/TiNPls).

Table SI 3. The percentage of fluorescence quenching and autofluorescence of different concentrations of TiO_2 nanoparticles (TiNPs), titanate nanotubes (TiNTs), TiO_2 nanowires (TiNWs), TiO_2 nanoplates (TiNPls), and precipitates formed after 60 min reaction time in the control system (calcium deficient hydroxyapatite, CaDHA) and in the presence of 100 mg L^{-1} TiO_2 nanoparticles (CaDHA/TiNPs), nanotubes (CaDHA/TiNTs), nanowires (CaDHA/TiNWs), and nanoplates (CaDHA/TiNPls).

Table SI 4. The results of optical interference assay and adsorption assay on different concentrations of TiO_2 nanoparticles (TiNPs), titanate nanotubes (TiNTs), TiO_2 nanowires (TiNWs), TiO_2 nanoplates (TiNPls), and precipitates formed after 60 min reaction time in the control system (calcium deficient hydroxyapatite, CaDHA) and in the presence of 100 mg L^{-1} TiO_2 nanoparticles (CaDHA/TiNPs), nanotubes (CaDHA/TiNTs), nanowires (CaDHA/TiNWs), and nanoplates (CaDHA/TiNPls) in hemolysis test.

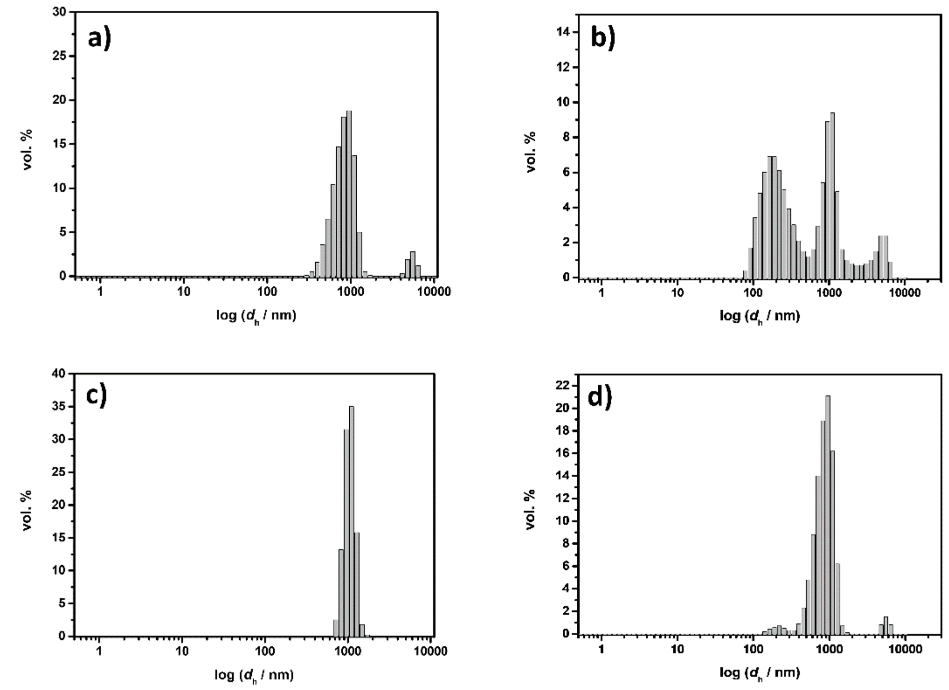

Figure SI 1. Volume size distributions of TiNPls (a,b) and TiNWs (c,d) in c-SBF (a,c) and anionic reactant solution (*c* (Na_2HPO_4) = 8 · 10^{-3} mol dm^{-3}, pH_{init} = 7.4, ϑ = (25.0 ± 0.1) °C.

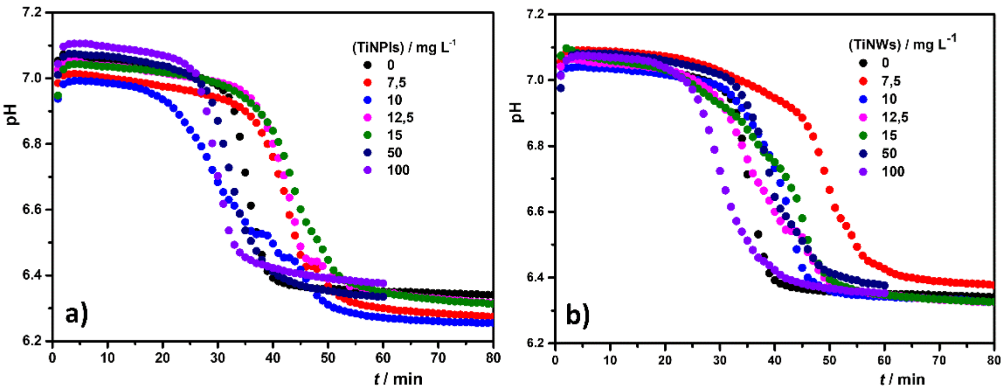

Figure SI 2. Representative pH *vs* time curves obtained in the control system (without TiO_2 nanomaterials) and in the presence of different concentrations of TiO_2 a) nanoplates (TiNPls) and b) nanowires (TiNWs). c ($CaCl_2$) = c (Na_2HPO_4) = $4 \cdot 10^{-}$ mol dm^{-3}, pH_{init} = 7.4, ϑ = (25 ± 0,1) °C.

Table SI 1. Average induction time obtained from pH *vs* time curves from 3 measurements with standard deviations in the control system (without TiO_2 nanomaterials) and in the presence of different concentrations of TiO_2 nanoplates (TiNPls) and nanowires (TiNWs). c ($CaCl_2$) = c (Na_2HPO_4) = $4 \cdot 10^{-3}$ mol dm^{-3}, pH_{init} = 7.4, ϑ = (25.0 ± 0.1) °C.

γ / mg L^{-1}	t_{ind} / min	
	TiNPls	TiNWs
0	29.4 ± 3.2	
7.5	36.4 ± 2.0	36.4 ± 4.0
10.0	35.0± 4.8	33.7 ± 2.4
12.5	35.8 ± 2.7	24.1 ± 4.5
15.0	36.1 ± 1.0	36.9 ± 2.3
50.0	27.1 ± 0.6	29.2 ± 1.34
100.0	25.3 ± 0.9	23.2 ± 1.6

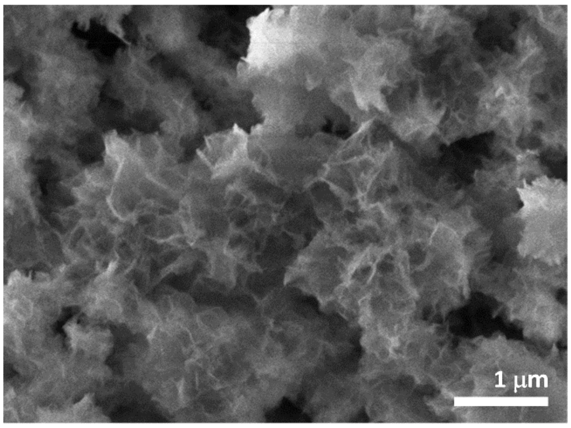

Figure SI 3. SEM micrographs of the calcium deficient hydroxyapatite (CaDHA) formed in control system after 60 min reaction time. *c* ($CaCl_2$) = *c* (Na_2HPO_4) = 4 · 10^{-3} mol dm^{-3}, pH_{init} = 7.4, ϑ = (25 ± 0.1) °C.

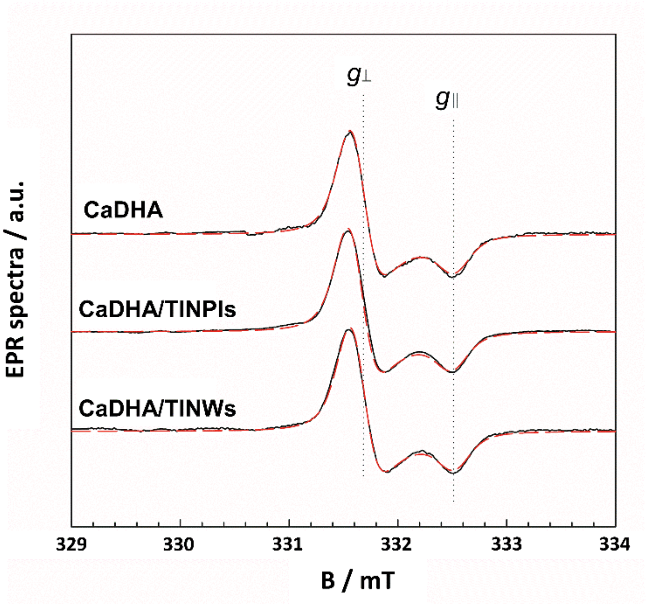

Figure SI 4. Simulated (red) and experimental (dark) EPR spectra of gamma irradiated precipitates formed after 60 min reaction time in the control system (calcium deficient hydroxyapatite, CaDHA) and in the presence of 100 mg L^{-1} TiO_2 nanoplates (CaDHA/TiNPls), and nanowires (CaDHA/TiNWs). Extracted spectral parameters are listed in Table SI 2.

Table SI 2. EPR spectral parameters extracted by simulation of experimental spectra according to Figures 8 and SI 4 for precipitates formed after 60 min reaction time in the control system (calcium deficient hydroxyapatite, CaDHA) and in the presence of 100 mg L^{-1} TiO_2 nanoplates (CaDHA/TiNPls), and nanowires (CaDHA/TiNWs).

Sample	g⊥	g‖	ΔHGpp*	ΔHLpp*
**CaDHA**	2.0025	1.9972	0.186	0.125
**CaDHA/TiNPls**	2.0025	1.9971	0.186	0.127
**CaDHA/TiNWs**	2.0026	1.9972	0.186	0.126

*The determined line widths refer to peak-to-peak Gaussian, ΔH_{Gpp} and Lorentzian, ΔH_{Lpp} line shape

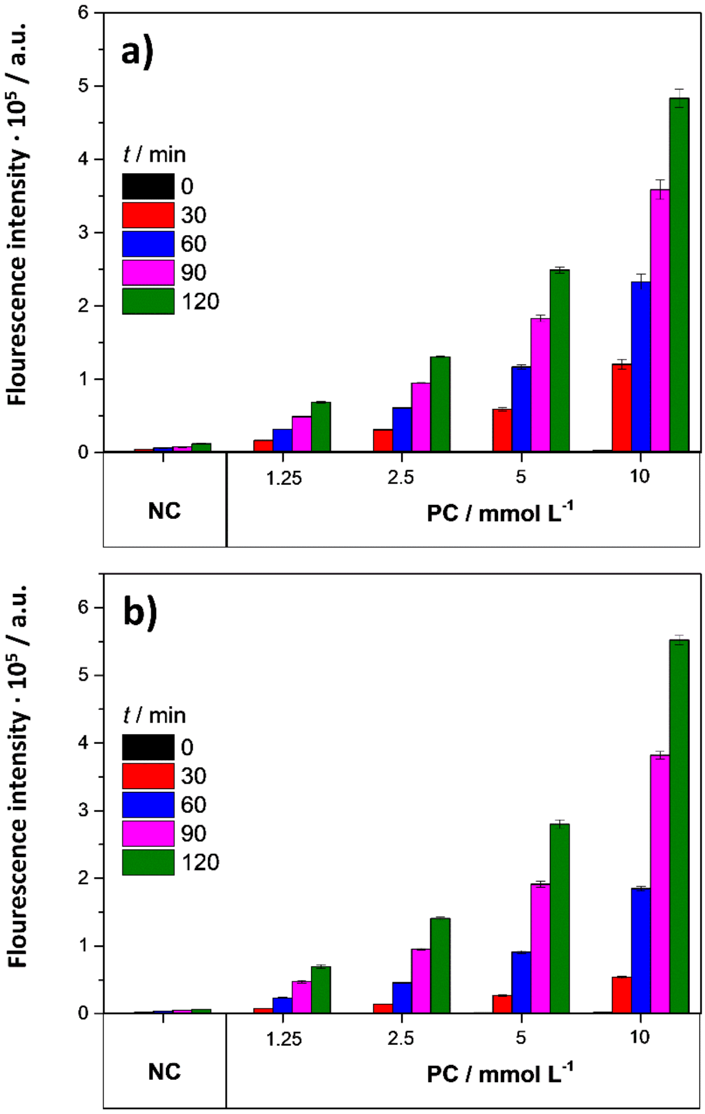

Figure SI 5. The intensity of DCF fluorescence after incubation in PBS with different concentrations of SIN-1 (NC – negative control; PC – positive control) used for comparison with a) TiO_2 nanoparticles (TiNPs), titanate nanotubes (TiNTs), TiO_2 nanowires (TiNWs), and TiO_2 nanoplates (TiNPls), and b) precipitates formed after 60 min of reaction time in the control system (calcium deficient hydroxyapatite, CaDHA) and in the presence of 100 mg L^{-1} TiO_2 nanoparticles (CaDHA/TiNPs), nanotubes (CaDHA/TiNTs), nanowires (CaDHA/TiNWs), and nanoplates (CaDHA/TiNPls).

Table SI 3. The percentage of fluorescence quenching and autofluorescence of different concentrations of TiO_2 nanoparticles (TiNPs), titanate nanotubes (TiNTs), TiO_2 nanowires (TiNWs), TiO_2 nanoplates (TiNPls), and precipitates formed after 60 min reaction time in the control system (calcium deficient hydroxyapatite, CaDHA) and in the presence of 100 mg L^{-1} TiO_2 nanoparticles (CaDHA/TiNPs), nanotubes (CaDHA/TiNTs), nanowires (CaDHA/TiNWs), and nanoplates (CaDHA/TiNPls).

.

**Nanomaterial**	**Concentration / mg L^{-1}**	**% quenching**	**% autofluorescence**
**TiNPs**	10	0%	102%
	100	5%	81%
	500	11%	86%
**TiNTs**	10	1%	101%
	100	11%	73%
	500	14%	67%
**TiNWs**	10	5%	227%
	100	23%	68%
	500	31%	75%
**TiNPIs**	10	1%	106%
	100	3%	97%
	500	6%	81%
**CaDHA**	10	4%	106%
	100	16%	71%
	500	21%	32%
**CaDHA/TiNPs**	10	1%	117%
	100	17%	53%
	500	31%	17%
**CaDHA/TiNTs**	10	1%	113%
	100	12%	71%
	500	27%	55%
**CaDHA/TiNWs**	10	5%	99%
	100	26%	31%
	500	30%	14%
**CaDHA/TiNPls**	10	5%	104%
	100	15%	59%
	500	32%	18%

Table SI 4. The results of optical interference assay and adsorption assay on different concentrations of TiO_2 nanoparticles (TiNPs), titanate nanotubes (TiNTs), TiO_2 nanowires (TiNWs), TiO_2 nanoplates (TiNPls), and precipitates formed after 60 min reaction time in control system (calcium deficient hydroxyapatite, CaDHA) and in the presence of 100 mg L^{-1} TiO_2 nanoparticles (CaDHA/TiNPs), nanotubes (CaDHA/TiNTs), nanowires (CaDHA/TiNWs), and nanoplates (CaDHA/TiNPls) in hemolysis test.

Material	Concentration (mg/L)	% absorbance (optical interference test)	% Hb recovery (adsorption test)
**Blank (PBS)**		100.00 ± 0.79%	100.00 ± 2.47%
**Negative control (PEG)**		N.A.	N.A.
**Positive control (Triton)**		N.A.	N.A.
**TiNPs**	10	98.73 ± 10.06%	87.21 ± 5.49%
	100	88.37 ± 10.40%	89.04 ± 5.61%
	500	100.61 ± 2.42%	98.30 ± 2.22%
**TiNTs**	10	96.30 ± 0.60%	97.78 ± 2.30%
	100	96.42 ± 6.39%	100.09 ± 1.82%
	500	107.33 ± 5.72%	98.99 ± 0.02%
**TiNWs**	10	97.93 ± 4.93%	106.27 ± 7.25%
	100	105.51 ± 3.68%	104.00 ± 1.76%
	500	101.38 ± 3.17%	99.61 ± 1.11%
**TiNPIs**	10	96.49 ± 4.20%	95.73 ± 0.45%
	100	100.67 ± 7.02%	94.44 ± 7.52%
	500	94.36 ± 7.91%	100.15 ± 2.92%
**CaDHA**	10	98.96 ± 0.58%	107.88 ± 9.39%
	100	101.49 ± 2.18%	104.46 ± 5.78%
	500	100.98 ± 0.88%	104.52 ± 5.04%
**CaDHA/TiNPs**	10	99.72 ± 1.14%	85.72 ± 7.00%
	100	108.46 ± 7.31%	99.71 ± 2.68%
	500	101.44 ± 2.15%	97.09 ± 1.66%
**CaDHA/TiNTs**	10	99.93 ± 1.12%	93.14 ± 4.16%
	100	102.63 ± 1.66%	94.33 ± 5.62%
	500	106.04 ± 4.64%	98.04 ± 0.55%
**CaDHA/TiNWs**	10	100.61 ± 1.52%	106.14 ± 3.21%
	100	104.66 ± 5.03%	95.19 ± 1.80%
	500	111.54 ± 9.69%	101.74 ± 0.68%
**CaDHA/TiNPls**	10	100.73 ± 0.93%	107.91 ± 5.07%
	100	107.18 ± 1.65%	92.96 ± 1.91%
	500	104.49 ± 1.93%	106.54 ± 5.17%
